# Endovascular treatment of an asymptomatic hepatic artery aneurism: case report

**DOI:** 10.1590/1677-5449.200123

**Published:** 2021-08-13

**Authors:** João Lucas O’Connell, Lucas Antônio Oliveira Faria, Marcela Gomes de Souza, Gabriel Alves Meneses, Alice Mirane Malta Carrijo

**Affiliations:** 1 Universidade Federal de Uberlândia – UFU, Faculdade de Medicina – FAMED, Uberlândia, MG, Brasil.

**Keywords:** hepatic aneurysm, visceral aneurysm, endovascular treatment

## Abstract

We report a case of an asymptomatic, 77-year-old, male patient with arterial hypertension and no other comorbidities or risk factors for coronary disease. During a routine abdominal ultrasound examination, he was diagnosed with a hepatic vascular mass with an approximate diameter of 5 cm. Abdominal computed angiotomography was requested, showing an aneurysm of the hepatic artery, with maximum diameter of up to 5.2 cm, longest longitudinal axis of 7.2 cm, and a maximum true lumen caliber of 3.0 cm. We opted for endovascular aneurysm repair with implantation of three sequential Lifestream covered vascular stents (7x58mm, 8x58mm, and 8x37mm), successfully diverting the flow through the stents and excluding the aneurysm. The patient remains asymptomatic and free from clinical complications 2 years after the procedure. Control examinations with arterial duplex ultrasound 6 and 12 months after the procedure showed good flow through the stents with no leakage into the aneurysmal sac.

## INTRODUCTION

Aneurysms of the visceral arteries (AVA) and pseudoaneurysms of the visceral arteries (PVA) are defined as arterial dilatations involving the celiac, superior mesenteric, or inferior mesenteric arteries and their branches. Aneurysms of the visceral arteries and PVA are relatively rare, leading to a low level of suspicion, although aneurysms of the splenic artery and aneurysms of the hepatic artery (AHA) are the most frequent types.^1^^-^3 Aneurysms of the visceral arteries were described for the first time in 1903 by the anatomist James Wilson. Known etiologies include atherosclerosis, abdominal trauma, surgical procedures, degenerative diseases, infections, connective tissue vascular disease, and congenital anomalies.^4^ Delayed diagnosis and a high propensity for rupture can sometimes result in severe hemorrhages. They can be detected by angiography, computed tomography angiography, or ultrasonography.^4^^-^6 The objective of treatment is to exclude the aneurysm sac from the circulation, while preserving distal flow, which can be accomplished via conventional surgical approaches or using endovascular techniques.^7^ We report the case of an asymptomatic hypertensive male patient with no other comorbidities or risk factors for coronary disease or peripheral arteriopathy who was diagnosed with AHA as an incidental finding of routine abdominal ultrasonography, enabling timely treatment by endovascular aneurysm repair, with sequential implantation of three covered vascular stents. The Research Ethics Committee approved this study (decision number 4.842.942).

## CASE DESCRIPTION

**Figure 1:**
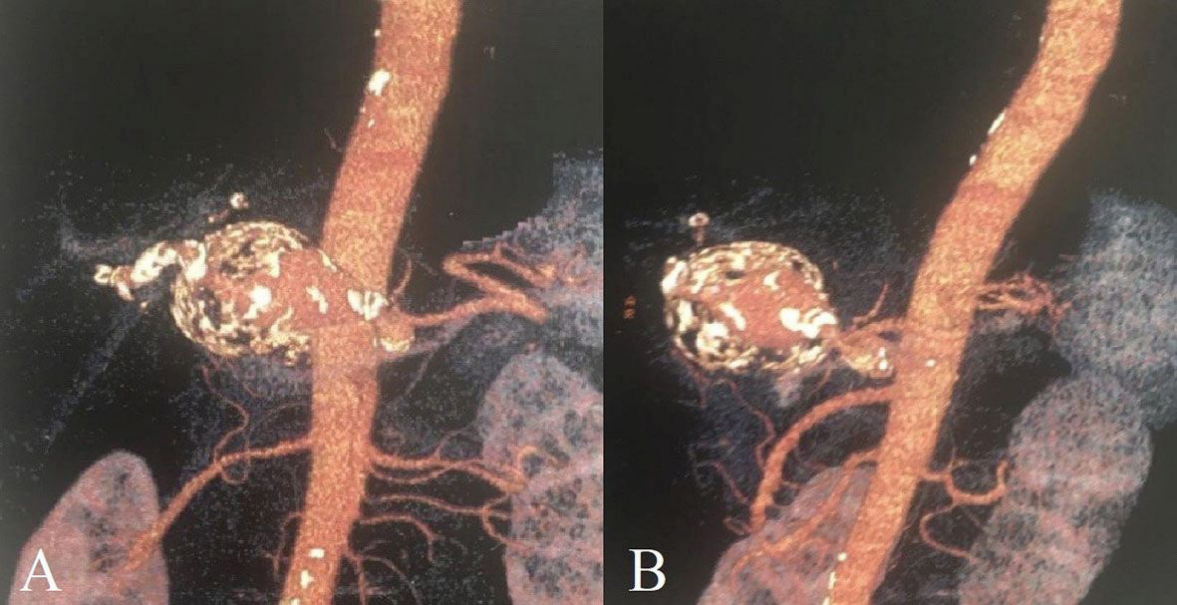
Abdominal angiotomography **A** and **B –** Presence of aneurysmal dilatation of the common hepatic artery.

The patient was a 77-year-old, hypertensive, white, Brazilian male with no other comorbidities or risk factors for coronary disease or peripheral arteriopathy. Routine abdominal ultrasonography was performed for evaluation of hepatic steatosis, identifying a mass in the hepatic region, probably of a vascular origin. Abdominal angiotomography was ordered, showing two areas of aneurysmal dilatation of the hepatic artery. The larger dilation was more proximal to the celiac trunk, with a length of around 7.2 cm along its longest longitudinal axis, a maximum caliber of 5.2 cm, and a maximum true lumen caliber of 3.0 cm. The second dilatation was closer to the hepatic hilum, with a length of around 2.0 cm, maximum caliber of 2.0 cm, and a small mural thrombus (Figure 1).

**Figure 2:**
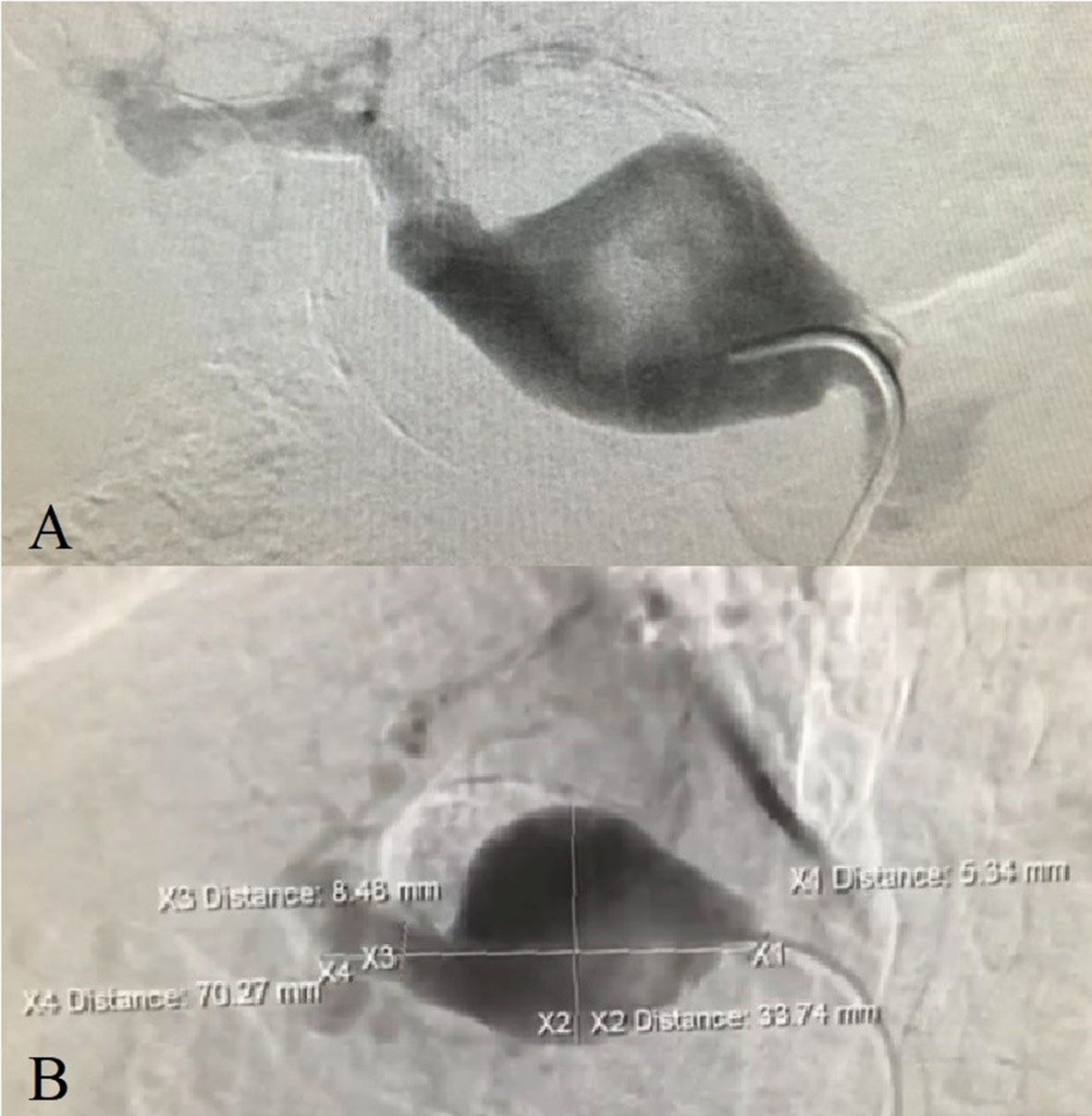
Arteriography. **A** and **B –** Presence of aneurysm of the common hepatic artery. Note the larger estimates of diameters and lengths than described in the abdominal angiotomography.

**Figure 3:**
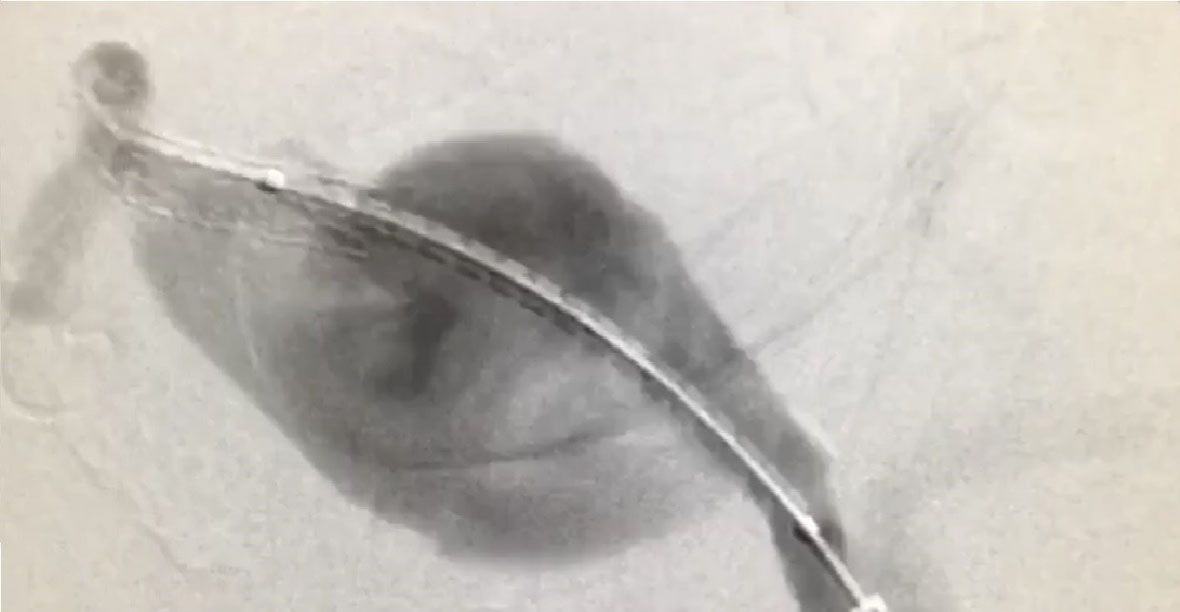
Arteriography showing positioning for deployment of the second sequential Lifestream^®^ stent overlapping the first stent deployed in the hepatic artery.

**Figure 4:**
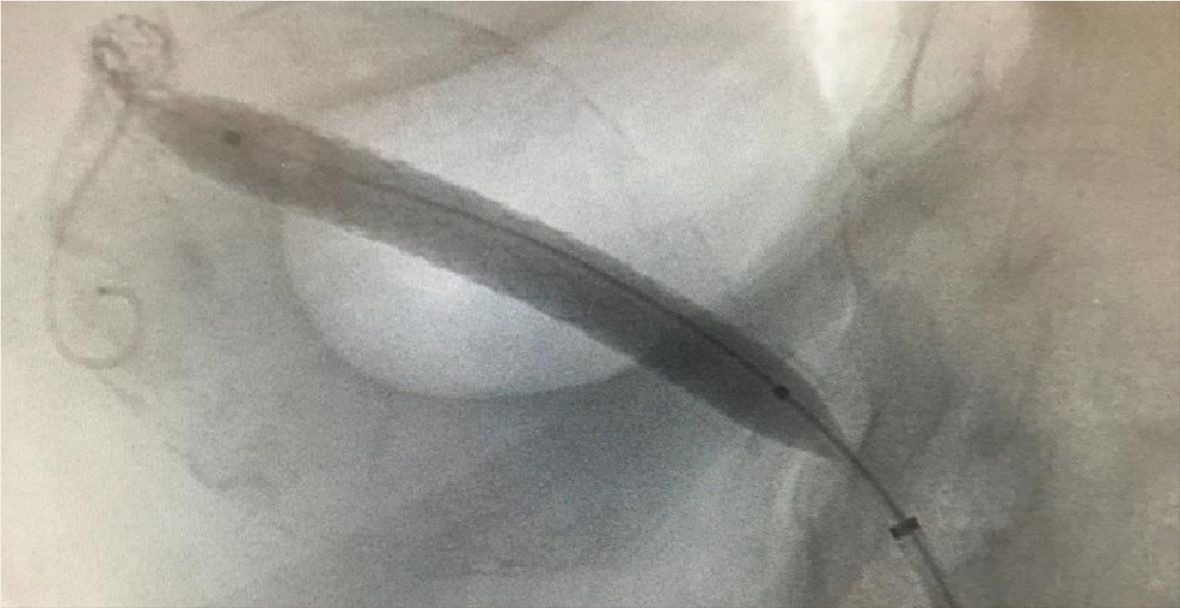
After expansion of the three stents using an 8x58 mm balloon.

**Figure 5:**
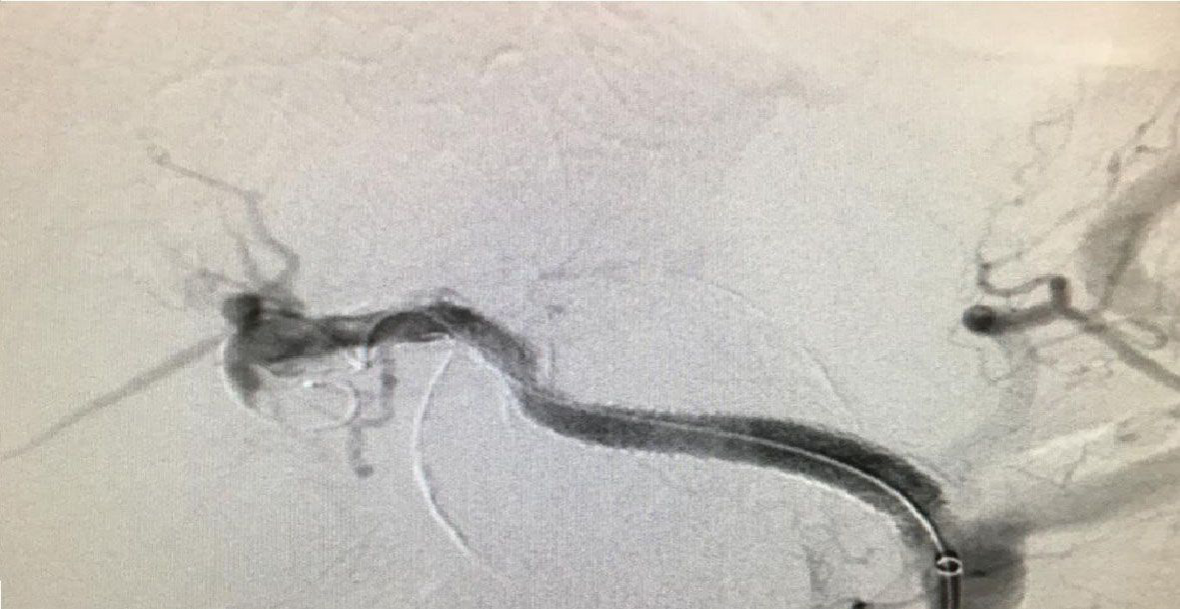
Final arteriography showing adequate placement of the three stents with flow diverted through the covered stents, excluding the aneurysm.

The management approach chosen comprised digital subtraction arteriography with simultaneous endovascular aneurysm repair using three Lifestream^®^ (Clearstream Technologies Ltd., Ireland) vascular covered stents, sizes 7x58 mm, 8x58 mm, and 8x37 mm, implanted sequentially, partially overlapping, with the aid of an HFAN 7F, valved introducer sheath and 0.35” Roadruner^®^ (Cook Medical Group, Indiana, United States) hydrophilic guidewire followed by an Amplatz Stiff^TM^ (Boston Scientific Corporation, Massachusetts, United States) hydrophilic guidewire up to the distal segment of the hepatic artery, to guide the stents as they were advanced (Figure 2). The procedure was performed successfully, achieving exclusion of the aneurysm (Figures 3, 4, and 5). The patient remained asymptomatic, with adequate flow through the stents shown on two follow-up ultrasound examinations, at 6 months and 1 year, and free from leaks into the aneurysm, which was still thrombosed 2 years after treatment.

## DISCUSSION

A true arterial aneurysm is defined as dilatation to at least one and half times the diameter of the vessel that is permanent and local and involves all three layers of the vessel wall. In contrast, a pseudoaneurysm is a local arterial rupture of tunica intima and tunica media, but which remains covered by adventitia or perivascular tissue.^8^ Aneurysms of the visceral arteries are rare conditions, but when they do occur the splenic artery is the most frequently involved, followed by the hepatic artery (20% of all AVA).^1^^-^3

In 80% of AHA, the location is extrahepatic.^4^ Of these, 60% involve the common hepatic artery, 30% the right hepatic artery and 5% the left hepatic artery.^9^ Incidence peaks between 50 and 60 years of age and they are twice as common in men.^3^^,^^4^^,^^10^ It is believed that they occur in 0.002% of the population.^11^

Hepatic artery aneurysms may remain asymptomatic for long periods of time and be diagnosed incidentally or in autopsies.^1^^-^3^,^^11^^,^^12^ Clinical manifestations and physical examinations can thus be nonspecific, especially in cases with no hemodynamic repercussions.^8^ However, some patients will exhibit jaundice, abdominal masses, hypovolemia, or shock secondary to rupture or gastrointestinal bleeding.^11^^,^^13^^,^^14^

Delayed diagnosis can lead to rupture of the vessel, hemorrhage, and risk of death.^5^ It is estimated that the risk of rupture varies in the range of 20 to 80% and these aneurysms very often rupture into the biliary tree, causing biliary colic, gastrointestinal hemorrhage, and jaundice.^3^^,^^15^^,^^16^ Rupture is a clinical entity that is associated with high morbidity and mortality unless treated promptly.^5^^,^^11^ The lethality of rupture is estimated at 21%.^15^^,^^16^

Angiography is considered the first-choice diagnostic method, because it shows the aneurysm’s size, shape, and location, enabling treatment planning, and will also delineate any collateral circulation.^4^^,^^6^ Use of computed tomography and ultrasound has made diagnosis more likely. Since AHA have a higher risk of rupture than aneurysms involving other sites, they are generally treated.^11^

On the subject of treatment of AHA, Society for Vascular Surgery (SVS) guidelines state that the recommendation for asymptomatic patients without significant comorbidities is to repair AHA with diameters exceeding 2.0 cm or growth exceeding 0.5 cm/year. In patients with comorbidities, open repair is recommended if the AHA is larger than 5.0 cm.^17^

The objective of treatment is to exclude the aneurysm sac from the circulation, preserving distal flow. If this is not possible, the aneurysmal artery is occluded. Both interventions can be accomplished via open approaches or with endovascular techniques and should be individualized to suit clinical status, surgical risk, aneurysm site, and vascular bed.^7^

Endovascular methods are becoming more widely used for treatment of AHA, especially in scheduled procedures, because of the high morbidity and mortality rates associated with open surgery. The principal advantage is lower invasivity, which is especially useful in high-risk patients.^18^ This is therefore the method initially recommended for all AHA in which it is anatomically viable, i.e., when it is possible to maintain arterial circulation to the liver.^17^

Interventional treatments for AHA can consist of percutaneous implantation of endoprostheses to achieve endovascular exclusion of the aneurysm, by occlusion of the hepatic artery (using embolization), or by open surgical repair of the arterial dilatation (surgical resection with graft interposition). With regard to the endovascular techniques, since maintenance of perfusion to the distal organ is important, a covered stent is always preferable to coil embolization.^17^ Reparative surgery is primarily indicated when there is no collateral vascularization to the hepatic segment involved.^19^

The Lifestream^®^ stents used in the case described were developed to treat patients with peripheral vascular disease. These devices are covered with a reinforced and durable material, expanded polytetrafluoroethylene, bonded to the external structure of the stent, made from Nitinol. Since they are flexible, these endoprostheses can be navigated along tortuous vessels and can fit more complex arterial anatomies. Moreover, since they are covered in polyethylene, they are useful for treatment of arterial aneurysms, since the covering stops blood from flowing through the mesh, as happens with uncovered stents, thereby minimizing the occurrence of endoleaks and enabling effective exclusion of the aneurysmal portion of the vessel.^20^

After implantation of a covered stent, the blood flow is diverted through the stent only, preventing the arterial flow from maintaining constant pressure against the dilated artery wall, which significantly reduces the likelihood of aneurysm rupture. The greatest disadvantage of the percutaneous technique is the risk of only partially excluding the aneurysm, which would result in leakage into the aneurysm and maintenance of the risk of rupture.^21^ Development of more refined techniques and devices may make this the preferred technique in the future.

In the present case, the choice to adopt an endovascular approach, implanting three partially overlapping Lifestream® stents in sequence was based on the anatomic viability, the possibility of maintaining patency of the hepatic artery, the greater risk of open surgery, and, primarily, the fact that the patient and his family expressed preference for the endovascular option. The treatment adopted in this case is in line with current intervention guidelines, which state that this approach is initially recommended for all AHA in which it is anatomically feasible, i.e. when it is possible to maintain arterial circulation to the liver.^17^ The patient remained asymptomatic, with flow detectable through the stents implanted in the hepatic artery on control ultrasound examinations and with no flow into the aneurysm sac, which remains thrombosed 2 years after treatment.
