# Supplementary material for: Endovascular treatment of an asymptomatic hepatic artery aneurism: case report
Source: J Vasc Bras. 2021 Aug 13;20:e20200123. doi: 10.1590/1677-5449.200123 (PMC8366405; doi:10.1590/1677-5449.200123)
Supplement: Translation

# Translation: Tratamento endovascular de aneurisma de artéria hepática assintomático: relato de caso

**Authors:** João Lucas O’Connell, Lucas Antônio Oliveira Faria, Marcela Gomes de Souza, Gabriel Alves Meneses, Alice Mirane Malta Carrijo

**Affiliations:** 1 Universidade Federal de Uberlândia – UFU, Faculdade de Medicina – FAMED, Uberlândia, MG, Brasil.

**Keywords:** aneurisma hepático, aneurisma visceral, tratamento endovascular

## Abstract

Relatamos o caso de um paciente masculino, 77 anos, portador de hipertensão arterial, sem outras comorbidades ou fatores de risco para coronariopatia. Apresentava-se assintomático e, durante exame de ultrassonografia abdominal de rotina, foi diagnosticada uma massa vascular hepática de, aproximadamente, 5 cm de diâmetro. Foi solicitada angiotomografia computadorizada abdominal, que evidenciou aneurisma de artéria hepática de até 5,2 cm de calibre máximo, 7,2 cm de extensão no maior eixo longitudinal e calibre máximo da luz verdadeira de 3,0 cm. Optou-se por realização de correção endovascular do aneurisma com implante de três endopróteses vasculares revestidas Lifestream 7x58 mm, 8x58 mm e 8x37 mm sequenciais, com sucesso, conseguindo-se direcionamento do fluxo pelas próteses, levando à exclusão do aneurisma. O paciente evoluiu assintomático, mesmo 2 anos após o implante, sem intercorrências clínicas. Controle com dúplex arterial, realizados 6 e 12 meses após o procedimento, evidenciaram bom fluxo pelas endopróteses, sem “*leak*” para o saco aneurismático.

## INTRODUÇÃO

Os aneurismas de artérias viscerais (AAV) e pseudoaneurismas de artérias viscerais (PAV) são definidos como dilatações arteriais que afetam as artérias celíaca, mesentérica superior ou inferior e seus ramos. Os AAV e PAV são relativamente raros, o que gera baixa suspeição, mas, entre eles, os aneurismas de artéria esplênica e aneurismas da artéria hepática (AAH) são os mais frequentes^1^^-^ 3. Os AAH foram descritos pela primeira vez em 1903, pelo anatomista James Wilson. Entre as etiologias conhecidas, destacam-se aterosclerose, trauma abdominal, procedimentos cirúrgicos, doenças degenerativas, infecções, doença vascular do colágeno e anomalias congênitas^4^. Por vezes, o atraso no diagnóstico e a alta tendência a ruptura podem ocasionar hemorragias graves. A detecção pode ser feita por meio de angiografia, angiotomografia computadorizada ou ultrassonografia^4^^-^ 6. O tratamento objetiva excluir o saco aneurismático da circulação, preservando o fluxo distal, e pode ser realizado por abordagem cirúrgica ou por terapia endovascular^7^. Relata-se um caso de um paciente do sexo masculino, hipertenso, sem outras comorbidades ou fatores de risco para coronariopatia ou para arteriopatia periférica, assintomático, diagnosticado incidentalmente com um AAH por ultrassonografia abdominal de rotina, fato que possibilitou tratamento em tempo hábil por meio da correção endovascular do aneurisma com implante de três endopróteses vasculares revestidas sequenciais. O protocolo foi aprovado pelo Comitê de Ética de nossa instituição (parecer número 4.842.942).

## DESCRIÇÃO DO CASO

Paciente brasileiro, masculino, branco, 77 anos, hipertenso, sem outras comorbidades ou fatores de risco para coronariopatia ou para arteriopatia periférica. Realizou-se ultrassonografia abdominal de rotina para avaliação de esteatose hepática, e foi identificada massa em topografia hepática de provável origem vascular. Solicitou-se angiotomografia abdominal, a qual evidenciou dois focos de dilatação aneurismática da artéria hepática, sendo a maior dilatação mais proximal ao tronco celíaco, estendendo-se por cerca de 7,2 cm no maior eixo longitudinal, com calibre máximo de 5,2 cm e com luz verdadeira apresentando calibre máximo de 3,0 cm. A outra dilatação encontrava-se mais próxima do hilo hepático, se estendendo por cerca de 2,0 cm, com calibre máximo de 2,0 cm, de igual forma, apresentando pequeno trombo mural (Figura 1).

**Figura 1:** Angiotomografia de abdome. **A** e **B –** Presença de dilatação aneurismática de artéria hepática comum.

Optou-se por realização de arteriografia com subtração digital e simultânea correção endovascular do aneurisma com implante de três endopróteses vasculares revestidas tipo Lifestream^®^ (Clearstream Technologies LTD, Irlanda) 7x58 mm, 8x58 mm e 8x37 mm sequenciais e parcialmente sobrepostas, com auxílio de bainha valvada tipo HFAN 7F, com auxílio de cordas guias 0,35” hidrofílicas tipo Roadruner^®^ (Cook Medical Group, Indiana, EUA) e depois Amplatz Stiff^TM^ (Boston Scientific Corporation, Massachusetts, EUA) até o segmento distal da artéria hepática para guiar a progressão das endopróteses (Figura 2). O procedimento foi realizado com sucesso, conseguindo-se excluir o aneurisma (Figuras 3, 4 e 5). O paciente permaneceu assintomático, com fluxo adequado através das endopróteses nas duas ultrassonografias sequenciais, com 6 meses e 1 ano após, e sem fluxo tipo “*leak*” para o aneurisma que permanece trombosado 2 anos após o tratamento.

**Figura 2:** Arteriografia. **A** e **B –** Presença de aneurisma de artéria hepática comum. Nota-se estimativa de diâmetros e extensões maiores do que aqueles descritos pela angiotomografia de abdome.

**Figura 3:** Arteriografia de posicionamento para o implante da segunda endoprótese tipo Lifestream^®^ sequencial e justaposta à primeira endoprótese implantada na artéria hepática.

**Figura 4:** Pós-expansão das três endopróteses com utilização de balão 8x58 mm.

**Figura 5:** Arteriografia final evidenciando posicionamento adequado das três endopróteses com direcionamento do fluxo pelas endopróteses recobertas, levando à exclusão do aneurisma.

## DISCUSSÃO

Um aneurisma arterial verdadeiro é definido como uma dilatação de, pelo menos, uma vez e meia o diâmetro do vaso, permanente e localizada, que envolva as três camadas de sua parede. De outro modo, um pseudoaneurisma é uma ruptura arterial localizada da íntima e camada média, mas que permanece revestido por adventícia ou tecido perivascular^8^. Os AAV são enfermidades raras e, quando ocorrem, a artéria esplênica é a mais atingida, seguida pela artéria hepática (20% do total)^1^^-^ 3.

A localização dos AAH, em 80% dos casos, é extra-hepática^4^. Desses, 60% são localizados na artéria hepática comum, 30% na artéria hepática direita e 5% na hepática esquerda^9^. Sua incidência é maior entre 50-60 anos de idade, sendo duas vezes mais comum em homens^3^^,^ 4 ^,^ 10. Acredita-se que ocorre em 0,002% da população^11^.

Os AAH podem não apresentar sintomas por um longo período de tempo, sendo diagnosticados incidentalmente ou em autópsias^1^^-^ 3 ^,^ 11 ^,^ 12. Desse modo, as manifestações clínicas podem ser inespecíficas, bem como o exame físico, especialmente nos casos sem repercussão hemodinâmica^8^. Entretanto, alguns pacientes apresentam icterícia, massas abdominais, hipovolemia ou choques secundários a ruptura ou sangramento gastrointestinal^11^^,^ 13 ^,^ 14.

O atraso no diagnóstico pode ocasionar ruptura do vaso, hemorragia e risco de morte^5^. Estima-se que o risco de ruptura varie entre 20 a 80% e, muitas vezes, ocorre para dentro da árvore biliar, ocasionando cólica biliar, hemorragia gastrointestinal e icterícia^3^^,^ 15 ^,^ 16. A ruptura é uma entidade clínica associada a alta morbimortalidade, a menos que seja tratada prontamente^5^^,^ 11. Estima-se que a ruptura tenha letalidade de 21%^15^^,^ 16.

A angiografia é considerada o principal método diagnóstico, pois evidencia o tamanho, a forma e a localização do aneurisma, permitindo uma programação terapêutica, além de delimitar a eventual presença de circulação colateral^4^^,^ 6. O uso da tomografia computadorizada e ultrassom ampliou a possibilidade do diagnóstico. Os AAH, por apresentarem maior risco de ruptura em comparação com outros locais, são comumente abordados^11^.

Em relação ao tratamento dos AAH, as Diretrizes da *Society for Vascular Surgery* (SVS) estabelecem que, para pacientes assintomáticos, sem comorbidades significativas, a recomendação é reparar os AAH com diâmetro maior que 2,0 cm ou com crescimento maior que 0,5 cm/ano. Já em pacientes com comorbidades, recomenda-se o reparo aberto, caso o AAH seja maior que 5,0 cm^17^.

O objetivo do tratamento é excluir o saco aneurismático da circulação, preservando o fluxo distal. Se não for possível, faz-se a oclusão da artéria aneurismática. Ambas podem ser feitas por abordagem aberta ou endovascular, necessitando de individualização de acordo com quadro clínico, risco cirúrgico, localização do aneurisma e leito vascular^7^.

A abordagem endovascular vem ganhando espaço no tratamento dos AAH, especialmente em procedimentos eletivos, devido à alta taxa morbimortalidade na cirurgia aberta. A principal vantagem é a menor invasividade, especialmente útil para pacientes de alto risco^18^. Sendo assim, essa abordagem é inicialmente recomendada para todos os AAH que sejam anatomicamente viáveis, ou seja, quando é possível manter a circulação arterial para o fígado^17^.

As abordagens intervencionistas no AAH podem ser realizadas através de implante percutâneo de endopróteses para exclusão endovascular do aneurisma, através da oclusão da artéria hepática (por embolização) ou através do reparo cirúrgico aberto da dilatação arterial (ressecção cirúrgica com interposição de enxerto). Em relação às técnicas endovasculares, uma vez que a manutenção da perfusão do órgão distal é importante, o stent recoberto é sempre preferível à embolização em espiral^17^. Os procedimentos reconstrutivos são indicados, principalmente, quando uma vascularização colateral para o segmento hepático relacionado não estiver presente^19^.

A endoprótese Lifestream^®^, utilizada no caso descrito, foi desenvolvida para o tratamento de pacientes que sofrem de doença vascular periférica. O dispositivo é revestido de um material reforçado e durável, politetrafluoretileno expandido, aderido a uma estrutura externa de stent de Nitinol. Por ser flexível, a endoprótese possibilita que vasos tortuosos sejam atravessados, e a endoprótese pode se adaptar a anatomias arteriais mais complexas. Além disso, por ser revestida de polietileno, ela se torna útil para o tratamento de aneurismas arteriais, uma vez que o recobrimento impede o fluxo de sangue através da malha metálica da endoprótese, como acontece nas próteses não recobertas, minimizando, assim, a presença de “*endoleaks*” e permitindo uma exclusão efetiva da porção aneurismática do vaso^20^.

Após o implante da endoprótese recoberta, o sangue passa a ser direcionado apenas para o interior das endopróteses, impedindo que o fluxo arterial mantenha pressão constante sobre a parede arterial dilatada, o que diminui significativamente a chance de ruptura do aneurisma. O risco de exclusão apenas parcial do aneurisma, que implicaria em “*leak*” para o interior do aneurisma e manutenção do risco de ruptura, é a maior desvantagem da técnica percutânea^21^. O desenvolvimento de técnicas e dispositivos mais refinados pode tornar essa técnica a preferida futuramente.

No presente caso, a escolha pela abordagem endovascular com implante de três endopróteses Lifestream®️ em sequência e parcialmente sobrepostas foi feita pela viabilidade anatômica, pela possibilidade de manutenção da perviedade da artéria hepática, pelo maior risco da cirurgia aberta e, principalmente, pela preferência do paciente e da família pela proposta de abordagem endovascular. A conduta adotada para o caso está de acordo com as diretrizes atuais de intervenção, que estabelecem que “essa abordagem é inicialmente recomendada para todos os AAH que sejam anatomicamente viáveis, ou seja, quando é possível manter a circulação arterial para o fígado”^17^. O paciente permaneceu assintomático, com fluxo presente através das endopróteses implantadas em artéria hepática nas ultrassonografias sequenciais e sem fluxo para o saco aneurismático, o qual permanece trombosado dois anos após o tratamento.
